# Fluid-Attenuated Inversion Recovery Vascular Hyperintensity in Cerebrovascular Disease: A Review for Radiologists and Clinicians

**DOI:** 10.3389/fnagi.2021.790626

**Published:** 2021-12-16

**Authors:** Lichuan Zeng, Jinxin Chen, Huaqiang Liao, Qu Wang, Mingguo Xie, Wenbin Wu

**Affiliations:** ^1^Department of Radiology, Hospital of Chengdu University of Traditional Chinese Medicine, Chengdu, China; ^2^Department of Geriatrics, Hospital of Chengdu University of Traditional Chinese Medicine, Chengdu, China; ^3^Department of Ultrasound, Hospital of Chengdu University of Traditional Chinese Medicine, Chengdu, China

**Keywords:** fluid-attenuated inversion recovery, FLAIR vascular hyperintensity, collateral circulation, transient ischemic attack, stroke

## Abstract

Neuroradiological methods play important roles in neurology, especially in cerebrovascular diseases. Fluid-attenuated inversion recovery (FLAIR) vascular hyperintensity (FVH) is frequently encountered in patients with acute ischemic stroke and significant intracranial arterial stenosis or occlusion. The mechanisms underlying this phenomenon and the clinical implications of FVH have been a matter of debate. FVH is associated with large-vessel occlusion or severe stenosis, as well as impaired hemodynamics. Possible explanations suggested for its appearance include stationary blood and slow antegrade or retrograde filling of the leptomeningeal collateral circulation. However, the prognostic value of the presence of FVH has been controversial. FVH can also be observed in patients with transient ischemic attack (TIA), which may have different pathomechanisms. Its presence can help clinicians to identify patients who have a higher risk of stroke after TIA. In this review article, we aim to describe the mechanism and influencing factors of FVH, as well as its clinical significance in patients with cerebrovascular disease.

## Introduction

Cerebrovascular diseases are harmful to human life because they have high mortality and disability rates. Fluid-attenuated inversion recovery (FLAIR) is widely used for the diagnosis of various intracranial diseases and is now recommended as a part of the routine protocol for magnetic resonance imaging (MRI) of stroke. FLAIR vascular hyperintensity (FVH) was first described in 1999 in a series of patients with acute stroke and subacute stroke (Cosnard et al., 1999). This finding has also been termed “hyperintense vessels on FLAIR,” the “hyperintense vessel sign,” and the “ivy sign” in the literature (Girot et al., 2007; Hacein-Bey et al., 2014; Lee et al., 2016; Nam et al., 2017; Grosch et al., 2020). FVHs are defined as focal, serpentine, or linear hyperintensities that are best visualized within the Sylvian fissure and are associated with large-vessel occlusion or stenosis (Lee et al., 2021). This neuroimaging sign has been observed not only in large-vessel stenoocclusive disease due to atherosclerosis but also in other diseases, such as Moyamoya disease and transient ischemic attack (TIA). FVH may be an important neuroimaging marker, and clinicians and radiologists should be trained to look for its presence.

FLAIR vascular hyperintensity is most likely to represent slow arterial blood flow, which is frequently encountered in patients with acute ischemic stroke. Slow blood flow is interpreted as slow retrograde flow in leptomeningeal collaterals or antegrade flow that corresponds to impaired hemodynamics (Nam et al., 2018). FVH may thus be of clinical significance in predicting cerebrovascular disease, especially when magnetic resonance angiography (MRA) is unavailable. However, the associations of FVH with clinical outcomes remain controversial. FVH may represent slow collateral flow that is effective in maintaining perfusion to penumbral regions, restricting the progression of ischemic lesions, and improving outcomes (Dong and Nao, 2019; Yuan et al., 2019; Derraz et al., 2021). On the other hand, FVH has been observed to correspond to perfusion deficits, larger lesions, and poor outcomes (Nam et al., 2017; Kim et al., 2019; Zhu et al., 2020; Wang et al., 2021). In this review article, we aim to review the current understanding of the physiology and clinical significance of FVH. This underappreciated neuroimaging sign may have important clinical implications.

## Mechanism and Incidence of FLAIR Vascular Hyperintensity

**FIGURE 1:**
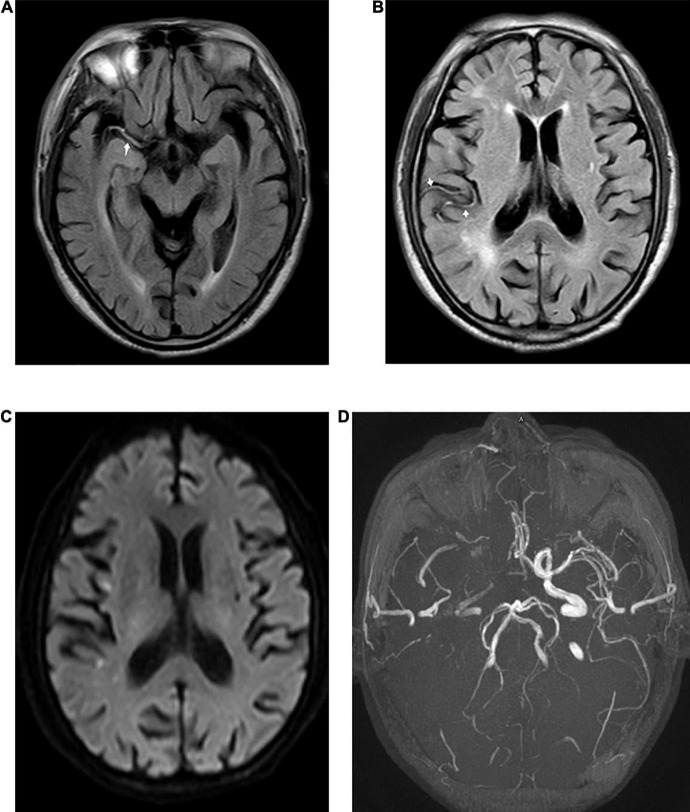
Fluid-attenuated inversion recovery (FLAIR) vascular hyperintensity (FVH) detected in a 73-year-old male patient with acute stroke. Proximal FVH in the Sylvian fissure [**(A)**, arrow] and distal FVH [**(B)**, arrow] in the right temporal lobe were detected in FLAIR images. Diffusion-weighted imaging (DWI) **(C)** shows acute ischemic infarction in the territory of the right middle cerebral artery (MCA). A time-of-flight magnetic resonance imaging (MRI) sequence **(D)** shows the right MCA and an occlusion of the internal carotid artery (ICA).

**FIGURE 2:**
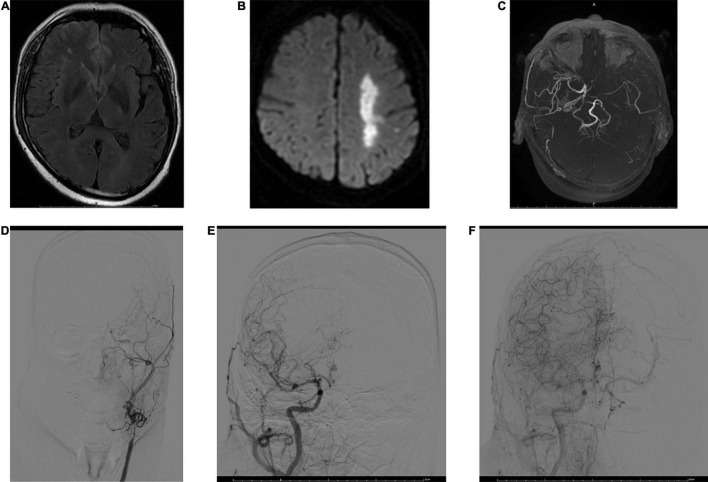
MRI and digital subtraction angiography (DSA) images obtained 3 h after stroke onset in a 53-year-old male. FVHs were observed at the left temporooccipital junction **(A)**. DWI shows acute infarction in the territory of the left MCA **(B)**. FVHs located beyond the DWI lesions, indicating an FVH–DWI mismatch. Magnetic resonance angiography (MRA) **(C)** shows near-occlusion of the left MCA and ICA. A DSA image obtained soon afterward demonstrates stenosis of the left common carotid artery and occlusion of the ICA **(D)**. Angiograms of the right common carotid artery in anteroposterior view in the early arterial phase **(E)** and late arterial phase **(F)** show good retrograde filling of the leptomeningeal collateral circulation in the left hemisphere.

The exact pathophysiology of FVH has not yet been clearly defined, but its associations with acute large-artery occlusion and chronic arterial stenosis have been widely accepted. FVHs are classified into distal and proximal FVHs according to their extent and location (Figure 1), which have different clinical implications (Azizyan et al., 2011). Distal FVH is defined as FVH that is present in the M3 and/or distal segments of the MCA (Yoshioka et al., 2013; Nam et al., 2017). Proximal FVH is defined as FVH that extends only within the territories of the M1 and/or M2 segments of the MCA (Shin et al., 2020; Li et al., 2021). Initially, some authors linked FVH to intraluminal thrombus, whereas other studies suggested that FVH might reflect slow arterial blood flow rather than intraluminal thrombus (Sanossian et al., 2009). FVH is associated with the leptomeningeal collateral circulation in arterial occlusive lesions. Stationary blood and slow antegrade flow have been suggested as possible explanations for proximal FVH. Slow retrograde collateral circulation has been regarded as the mechanism of distal FVH (Figure 2). FVHs are frequently detected in patients with acute cerebral infarction accompanied by significant stenosis or arterial occlusion of the middle cerebral artery (MCA) and internal carotid artery (ICA). FVHs can also be observed in posterior cerebral artery territory, but it is much less reported than in MCA and ICA, for the anatomical characteristics of the PCA including a short and tortuous pathway compared with that of the MCA.

Previous studies have attributed the presence of FVH to large-artery disease, leptomeningeal collateral flow, and local alterations in hemodynamics (Jiang et al., 2019; Bourcier et al., 2020; Derraz et al., 2021; Lee et al., 2021; Maruyama et al., 2021). This hypothesis has been corroborated by several observations that reported the presence of FVH together with large-vessel stenosis or occlusion. Alterations in hemodynamics, such as stationary blood and slow blood flow, often from the collateral circulation through leptomeningeal anastomoses, have been suggested to be the leading cause of FVH (Lee et al., 2021). Ahn et al. (2016) investigated the influence of differences in blood flow velocity on the presentation of FVHs. As the flow velocity increased, the signal intensity of FVHs decreased. This finding is consistent with the theory based on slow blood flow. Patients with cerebral infarction exhibited a higher frequency of FVH than patients with TIA, which suggests that the frequency of FVH might be correlated with the severity of ischemia.

FLAIR vascular hyperintensity has frequently been encountered in patients with acute ischemic stroke and significant intracranial arterial stenosis or occlusion, and it has also been observed in patients with chronic intracerebral arterial stenoocclusive disease. The frequency of FVHs has varied immensely between different studies. FVH signs on FLAIR have been reported to be observed in 45–100% of patients with acute ischemic stroke, primarily in strokes involving the MCA territory. Shin et al. (2020) showed that proximal FVH was observed in 71/105 (67.6%) patients who presented with hyperacute infarction of the MCA. A lower prevalence of FVH has been reported in distal occlusions and posterior strokes or when the onset-to-imaging time increased. One possible explanation for these results is the heterogeneity of the patients in the studies. A great many variables might affect the presentation of FVHs.

## Factors Influencing FLAIR Vascular Hyperintensities

Many factors may influence the presence and extent of FVHs, of which the blood flow velocity is regarded as the most important. A quantitative MRI study (Ahn et al., 2016) of a phantom using different flow velocities would help to define the exact range of flow velocities that cause FVH. The signal intensities of FVHs on conventional FLAIR were minimal at a flow velocity of 11.3 cm/s, but as flow velocities decreased the signal intensities increased. Hence, a decrease in blood flow velocity will result in an increase in signal intensity in the respective vessel on FLAIR imaging. On the other hand, Ahn et al. (2016) reported that MRI parameters, such as the echo time (TE) and flip angle (FA), and the periodically rotated overlapping parallel lines with enhanced reconstruction (PROPELLER) technique were independent factors that influenced the intensity of FVHs. As the TE increased, the signal intensity of FVHs decreased. In contrast, as the FA of the refocusing pulse increased, the intensity of FVHs increased, and the PROPELLER technique significantly increased the intensity of FVHs (Ahn et al., 2015).

The average time interval between symptom onset and imaging might be another important factor that influences the presence of FVH. Many studies have examined the time course of FVH in acute and subacute cerebral infarctions of the MCA territory, and the results showed that the frequency of FVH declined over time (Aoki et al., 2020). Maeda et al. (2001) reported that FVH was seen in 100% of examinations carried out less than 24 h after symptom onset but only 50% of examinations performed 10–13 days after symptom onset. The presence of FVH is associated with the time interval between stroke onset and MRI scanning. FVH can be a temporary phenomenon that commonly disappears within the first 24–36 h after stroke onset. FVH is recognized as a marker of slow blood flow induced by severe stenosis or occlusion of vessels. A previous study (Liu et al., 2011) suggested that there was a significant positive correlation between the intensity of FVH and the degree of stenosis of the M1 segment.

## Evaluation Methods

Several approaches have been proposed for investigating the extent of FVHs. Lee et al. (2009) graded distal FVHs as absent, subtle, and prominent. Subtle FVH was defined as FVH present over less than one third of the perfusion lesion, whereas prominent FVH was defined as FVH present over more than one third of the perfusion lesion. Olindo et al. (2012) reported a scoring system based on a rostrocaudal extension of FVH. They analyzed horizontal FLAIR images from the first appearance of the M1 segment of the MCA to the 10th image. On each slice, absence of FVH scored 0 points, whereas if one or more FVHs were recognized this scored 1 point. The resulting FVH scores thus ranged from 0 to 10 after 10 images were analyzed.

The Alberta stroke program early computerized tomography score (ASPECTS) was a widely used method for assessing computerized tomography scans in patients with acute ischemic stroke (Barber et al., 2000). The scores on ASPECTS for seven cortical areas (insula and M1–M6) were used to assign FVH scores according to the spatial distribution of MCA. The insula and M1–M3 were defined as corresponding to the level of the basal ganglia, and M4–M6 were defined as corresponding to the level of the ventricles immediately above the basal ganglia, as follows: M1, anterior cortex of MCA; M2, lateral cortex of MCA; M3, posterior cerebral cortex of MCA; and M4, M5, and M6, the anterior, lateral, and posterior MCA territories immediately superior to M1, M2, and M3, respectively. The FVH score on ASPECTS, which is based on the number of territories that are positive for FVH, is more widely accepted and applied in clinical practice.

## Clinical Applications

### Prediction of Arterial Stenosis or Occlusion

FLAIR vascular hyperintensities are most frequently identified in patients with persistent large-vessel stenosis or occlusion and acute ischemic stroke. FVH has demonstrated excellent diagnostic performance for the identification of large-vessel occlusion, especially in MCA and ICA. Many studies have assessed the accuracy of FVH for the confirmation and location of a large-vessel occlusion. They reported that FVH had excellent sensitivity (76–98%) and specificity (69.8–86%) for the identification of a large-vessel occlusion (Shin et al., 2020). In some FVH positive patients without large-vessel occlusion, atrial fibrillation was thought be an important factor. As for some FVH false negative patients, it is believed that these patients often have good collateral circulation and do not have slow blood flow conditions to form FVH signs.

FLAIR vascular hyperintensities frequently appear in patients with acute ischemic stroke and can, in fact, also be seen in TIA patients. Yoshioka et al. (2013) performed a retrospective analysis of TIA patients and analyzed the relationship between distal hyperintense vessels, severe large-artery stenosis or occlusion, and clinical presentation. They found that FVH was independently associated with severe large-artery stenosis or occlusion in TIA patients. FVH can be regarded as a marker of large-vessel occlusion, which is known to increase the short-term risk of stroke. The presence of FVH on initial FLAIR MRI may be a clue to the presence of persistent large-vessel stenosis or occlusion and the possibility of subsequent stroke. This sign is important, especially when MRA is unavailable to document the presence or absence of large-vessel stenosis or occlusion.

### Collaterals

The collateral vessels can maximize the odds of survival of a high volume of brain tissue by sustaining the ischemic penumbra. Most patients with acute stroke who have robust collaterals usually have better clinical outcomes. FVH in collaterals could serve as good evidence of hemodynamic impairment in patients with cerebral infarction and TIA. Liu et al. (2011) investigated the relationship between the location of FVH and the pattern of collateral flow by comparing findings on FLAIR images with those obtained by digital subtraction angiography. The results demonstrated that the location of FVH is an indicator of the pattern of collateral flow: FVH located within the Sylvian fissure mainly indicates antegrade collateral flow across the residual M1 segment; FVH present in the cerebral sulci at the temporooccipital junction usually represents retrograde leptomeningeal collateral flow from the anterior cerebral artery to the MCA; and FVH expanding to the cerebral sulci of the frontal and parietal lobes represents retrograde leptomeningeal collateral flow via the posterior cerebral artery to the MCA. FVH is a non-invasive indicator of both perfusion defects and collateral flow and has been evaluated in many studies of ischemic stroke. Some studies found that FVH represents insufficient collateralization and is associated with poor functional outcomes. However, other studies have reported that FVH is indicative of sufficient collateralization and has good prognostic value.

### Perfusion Abnormalities and FLAIR Vascular Hyperintensity–Diffusion-Weighted Imaging Mismatch

FLAIR vascular hyperintensities are common in patients with acute ischemic stroke and represent marked hemodynamic impairment and slow retrograde flow in the ischemic territory due to intracranial stenoocclusive disease. The presence of FVH in acute ischemic stroke may indicate cerebral hypoperfusion. The FVH sign is associated with larger lesion volumes on perfusion-weighted imaging (PWI) and mismatches between PWI and DWI volumes in acute stroke. Prominent or extended FVHs indicate large areas of salvageable tissue and greater potential benefits from recanalization. Patients in an FVH-positive group exhibited more severe hemodynamic impairment than those in an FVH-negative group (Gawlitza et al., 2014). A quantitative analysis of the perfusion parameters also revealed that perfusion was more severely compromised and widely disturbed in an FVH-positive group (Nomura et al., 2020).

The FVH score has been strongly associated with the area of hypoperfusion and the extent of prolongation of mean transit time, which suggests that the presence of FVH is representative of impaired cerebrovascular autoregulation. FVH can even sometimes be observed during the hyperacute phase of ischemic stroke. A previous study showed that patients with lower FVH scores were more likely to have smaller penumbras on computed tomography perfusion imaging and larger infarct volumes. The presence of FVH may be especially important in instances where MRA or perfusion imaging is not available or images are degraded by artifacts.

An FVH–DWI mismatch is considered to be present when FVH extends beyond the boundaries of the DWI cortical lesion (Figure 2). Legrand et al. (2015) reported that an FVH–DWI mismatch predicted the presence of a PWI–DWI mismatch with a sensitivity of 92% and a specificity of 64%. A PWI–DWI mismatch is thought to represent the ischemic penumbra or ischemia without permanent cellular damage. An FVH–DWI mismatch was associated with a smaller initial infarct and greater infarct growth after thrombolysis, even though the final infarcts remained smaller. Jiang et al. (2020a) also reported that an FVH–DWI mismatch group had smaller DWI volumes on admission and on follow-up but lower DWI volume growth than a group with no FVH–DWI mismatch.

Whether an FVH–DWI mismatch is a primary indicator of clinical outcomes in acute ischemic stroke is controversial. Jiang et al. (2020b) assessed the association between FVH–DWI mismatch and functional outcomes in patients with acute stroke receiving endovascular therapy. Patients with an FVH–DWI mismatch had higher FVH scores, smaller DWI volumes, and better functional outcomes than patients without an FVH–DWI mismatch. Wang et al. (2020) found that FVH–DWI mismatch was positively correlated with and independently associated with complete revascularization. They found that a group with good functional outcomes had higher FVH scores, higher FVH–DWI mismatch ratios, and higher complete revascularization ratios than a group with poor functional outcomes.

FLAIR vascular hyperintensity located beyond the boundaries of the DWI lesion reflects impaired yet viable tissue. This tissue recovers its function when recanalization is achieved, which explains why patients with an FVH–DWI mismatch are more likely to have favorable outcomes. PWI–DWI mismatch has been proposed for use in selecting patients with acute stroke for recanalization therapy. FVH–DWI mismatch provides an alternative to PWI–DWI mismatch for selecting patients as candidates for thrombectomy. FVH–DWI mismatch may rapidly identify patients with proximal occlusion who are most likely to benefit from recanalization (Legrand et al., 2016). It may also provide an alternative to PWI–DWI mismatch for identifying candidates for endovascular therapy (Legrand et al., 2019). FVH appearing in the early phase is associated with large-vessel occlusion, a higher National Institutes of Health Stroke Scale (NIHSS) score at admission, and a larger infarct core volume. Over time, FVH represents collateral blood supply to the arterial occlusion, saving more tissue in the ischemic penumbra, and improving the clinical prognosis.

### FLAIR Vascular Hyperintensity in Transient Ischemic Attack Patients

Transient ischemic attack is characterized by sudden focal brain dysfunction lasting less than 24 h, which is linked to cerebral or optic ischemia. Patients presenting with transient neurological symptoms have an increased risk of a subsequent stroke. Therefore, the clinical importance of early diagnosis and treatment of TIA should be emphasized to prevent the development of stroke. According to previous studies, the incidence of FVHs in TIA patients was markedly lower than in acute ischemic stroke and varied remarkably from 16 to 39.6% (Dong et al., 2017; Nam et al., 2018). Because some FVHs in TIA patients were proved to be transient and were correlated with symptom resolution, the prior reports may have underestimated the frequency of FVHs. Ding et al. (2020) reported that FVH was observed in up to 81.6% (31/38) of hospitalized TIA patients, who had severe stenosis or occlusions. On the another hand, they found that there was no significant difference in the degree of stenosis between FVH-negative and FVH-positive groups, which means that the severity of arterial stenosis did not predict the presence and extent of FVH.

FLAIR vascular hyperintensity has not been fully evaluated in patients with TIA in contrast to ischemic stroke. Two factors – arterial occlusion or stenosis and atrial fibrillation – have been reported to have significant and independent associations with FVH (de Figueiredo et al., 2017). It has been widely accepted that FVH is associated with the presence of significant large-artery stenosis or occlusion, which is an independent risk factor and has a high predictive value for stroke after TIA. FVH is associated with recurrent ischemic stroke events in patients with lesion-negative TIA (Nam et al., 2018). Atrial fibrillation has been specifically associated with transient FVH, and arterial occlusion or stenosis has been associated with persistent FVH. Kobayashi et al. (2013) reported that atrial fibrillation was more common than arterial occlusive lesions in FVH-positive patients. Transient FVH could be caused by early recanalization of emboli in patients with TIA.

**FIGURE 3:**
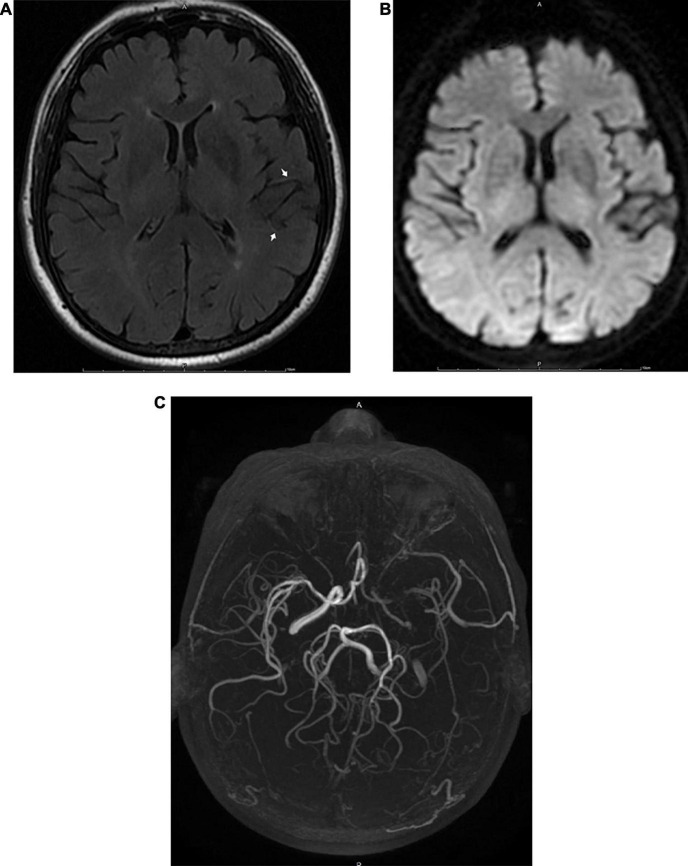
MRI images obtained 6 h after the appearance of the initial symptoms in a 68-year-old male patient with transient ischemic attack. A FLAIR image **(A)** shows FVH in the territory of the left MCA (arrow). There were no abnormalities on DWI **(B)**. Three-dimensional time-of-flight MRA **(C)** shows occlusion of the ipsilateral MCA and ICA.

FLAIR vascular hyperintensity can predict an oncoming acute ischemic stroke in the 30 days following a TIA (Dong et al., 2017). The occurrence of FVH in patients with TIA may be associated with subsequent ischemic stroke in the corresponding vascular territory. Predicting recurrent ischemic stroke after TIA is important in order to consider adequate strategies for managing patients with TIA. Persistent FVH has been associated with a mechanism of arterial occlusion and an increased risk of recurrent TIA and ischemic stroke (Figure 3). In contrast, transient FVH together with normal MRA findings has been linked to a mechanism of paroxysmal atrial fibrillation but not associated with an increased risk of stroke (Kobayashi et al., 2013). FVH may be of clinical significance in predicting ischemic stroke in a TIA setting, especially when MRA is unavailable.

### Association With Functional Outcomes

Many studies have focused on the association between FVHs and clinical outcomes in patients with ischemic stroke. However, the prognostic value of FVH findings is unclear. Some studies demonstrated that the presence of FVHs was associated with severe clinical impairments and poor functional outcomes, (Girot et al., 2007; Kufner et al., 2015; Dong et al., 2017; Nam et al., 2017; Zhu et al., 2020; Wang et al., 2021) whereas other studies indicated that FVHs were correlated with good collateral flow and favorable outcomes (Pérez et al., 2012; Dong and Nao, 2019; Yuan et al., 2019; Aoki et al., 2020; Jiang et al., 2020b; Wang et al., 2020; Zhou et al., 2020b; Derraz et al., 2021). In addition, some other studies proposed that FVH has different clinical significance under different conditions regarding prognostic meaning other than information regarding arterial occlusion (Kim et al., 2016, 2019; Liu et al., 2016; Sakuta et al., 2016; Li et al., 2018, 2021; Jiang et al., 2019; Shang et al., 2019; Zhou et al., 2020a). Studies with prediction of FVH for functional outcomes are summarized in Tables 1–3. Li et al. (2021) reported that high FVH scores in patients with acute stroke and occlusion or severe stenosis of the MCA tended to indicate severe clinical impairments and poor clinical outcomes, but subgroup analysis showed that high FVH scores represented favorable clinical outcomes in patients with occlusion of the MCA. The results indicated that the clinical significance of FVH differed between patients with severe stenosis of the MCA and patients with occlusion of the MCA.

**TABLE 1:** Studies with prediction of FVH for favorable functional outcome.

Author/Year	No. of patients	Mean Age (year) (range)	Standard for favorable functional outcome	Onset to imaging
Derraz et al. (2021)	85	92.4	90-day mRS ≤3	90-day mRS 0–3 group: 180 min 90-day mRS 4–6 group: 219 min
Wang et al. (2020)	72	69.69 (40–82)	3 months mRS score ≤2	Within 6 h
Jiang et al. (2020b)	59	FVH/DWI mismatch: 64.20 ± 14.97 No FVH/DWI mismatch: 71.28 ± 11.10	3 months mRS score ≤2	Within 6 h
Aoki et al. (2020)	72	76 (66–83)	Presence or absence of recanalization assessed by MRA or DSA	FVH(−) group: 9.0 h FVH(+) group: 2.6 h
Zhou et al. (2020b)	293	NA	90-day mRS ≤1	<4.5 h
Yuan et al. (2019)	68	Favorable: 61 (57.5–70.25) Unfavorable: 60 (50.25–64.25)	3 months mRS ≤2	Favorable group: 66 h Unfavorable group: 53.8 h
Dong and Nao (2019)	160	64.01 ± 11.81	90-day mRS ≤2	24.72 ± 16.24 h
Pérez et al. (2012)	70	66	Ischemia progression	Absent/Subtle/Prominent of FVH: 300/280/290 min

*FVH, FLAIR vascular hyperintensity; mRS, modified Rankin Scoring; NA, not available.*

**TABLE 2:** Studies with prediction of FVH for unfavorable functional outcome.

Author/Year	No. of patients	Mean Age (year) (range)	Standard for favorable functional outcome	Onset to imaging	Notes
Nam et al. (2017), Wang et al. (2021)	203	63.3 ± 10.2	mRS at discharge <2	NA	FVH after therapy
Girot et al. (2007), Zhu et al. (2020)	267	66.06 ± 11.76	90-day mRS ≤2	44.44 ± 16.48 h	
Dong et al. (2017), Wang et al. (2021)	154	63.0 ± 11.9	30-day follow-up acute ischemic stroke	Within 72 h	
Nam et al. (2017), Zhu et al. (2020)	325	69	Early neurological deterioration	Within 24 h	Distal FVH
Kufner et al. (2015), Dong et al. (2017)	62	71.4 ± 13.9	3 months mRS ≤2	Visible FHV on ≤4 Sections: 88.5 min Visible FHV on >4 Sections: 93.5 min	
Girot et al. (2007); Kufner et al. (2015)	30	64 (35–92)	1 month mRS ≤2	Within 12 h	Distal FVH

*FVH, FLAIR vascular hyperintensity; mRS, modified Rankin Scoring; NA, not available.*

**TABLE 3:** Studies with prediction of FVH for uncertain functional outcome.

Author/Year	No. of patients	Mean Age (year) (range)	Conclusion
Li et al. (2021)	282	66.66 ± 11.29	In patients with proximal MCA occlusion or stenosis ≥70%, a high FVH score represented severe clinical impairment and poor clinical outcomes In acute ischemic stroke patients with proximal MCA occlusion, a high FVH score represented favorable clinical outcomes
Zhou et al. (2020a)	3,577	NA	FVHs were not associated with functional outcome overall, but were significantly associated with better outcome in those receiving endovascular therapy
Jiang et al. (2019)	37	69.41 ± 12.51	The good functional outcome group had a higher FVH1 (before therapy) score and a lower FVH2 (after therapy) score than the poor functional outcome group
Shang et al. (2019)	459	NA	FVH is associated with unfavorable outcome within 6 h to 14 days of onset, while the wider distribution of distal FLAIR vascular hyperintensity may be favorable beyond 14 days of onset in MCA infarction
Kim et al. (2019)	112	67 (54–79)	For acute stroke patients who do not receive reperfusion therapy, prominent FVH may be independent predictors of an unfavorable outcome In the reperfusion therapy group, there was no association between prominent FVH and the clinical outcome
Li et al. (2018)	38	62.52 ± 13.61	FVH score showed no correlation with 90-day functional clinical outcome and was not sufficient as an independent predictor of short-term clinical outcome
Kim et al. (2016)	87	FVH(−): 70 (61.7–77.0) FVH(+): 71.5 (58.0–76.0)	FVH are associated with relatively severe clinical presentation and non-favorable prognosis in patients with cortical borderzone infarcts, but not in patients with internal borderzone infarcts
Liu et al. (2016)	101	66.2 ± 17.8	Higher FVH-ASPECTS measured outside the DWI lesion is associated with good clinical outcomes FVH-ASPECTS measured inside the DWI lesion was predictive of hemorrhagic transformation
Sakuta et al. (2016)	118	76 ± 9.7	Decrease of FVH after t-PA therapy predicts good outcome in patients receiving

*FVH, FLAIR vascular hyperintensity; MCA, middle cerebral artery; ASPECTS, alberta stroke program early computerized tomography score; NA, not available.*

FLAIR vascular hyperintensity has been recognized as a marker of collateral flow in ischemic stroke, but its relationship with outcomes is still controversial. There is little consensus on the interpretation of prognostic information from FVH studies, which should adopt an accepted standard as a prognostic assessment tool. In previous studies, the populations with stroke that were included were heterogeneous, especially in terms of the time to the initial MRI scan from symptom onset, and patients with proximal and distal FVHs were considered together, which potentially confounded tests for significance. Furthermore, studies used different prognostic assessment criteria, including the NIHSS score, Modified Rankin Scale score, and infarct volume on DWI.

The association between FVH and functional outcomes varies with time. Shang et al. (2019) reported that the symptom-to-imaging time might be an important factor when assessing the prognostic value of FVH. FVH is associated with unfavorable outcomes within 6 h to 14 days after onset, whereas a wider distribution of distal FVH may be favorable beyond 14 days after onset in infarction of the MCA. In the majority of studies that showed an association between FVH and good outcomes (Aoki et al., 2020; Jiang et al., 2020b; Wang et al., 2020; Zhou et al., 2020b; Derraz et al., 2021) the symptom-to-imaging time was less than 6 h. In contrast, in most of the studies that demonstrated an association between FVH and poor outcomes (Kim et al., 2016; Dong et al., 2017; Zhu et al., 2020) the symptom-to-imaging time was 12–24 h or longer. FVH might be an imaging marker of leptomeningeal collateral flow within 6 h of symptom onset or within the time window for reperfusion therapy. The extent of FVH may represent the volume of brain parenchyma at risk of ischemia, which could be saved by reperfusion therapy to reduce the final lesion size and improve functional outcomes. This may be the explanation why within this time interval patients with FVH may have better clinical outcomes than patients without FVH. On the other hand, when FVH occurs beyond the time window for reperfusion therapy, the presence of FVH may represent persistent occlusion of a vessel and impaired hemodynamics. Consequently, patients with FVH could be affected more by hemodynamic instability than patients without FVH. This difference might be associated with severe clinical impairments and unfavorable outcomes in patients with FVH during this time interval.

Liu et al. (2016) reported that the pattern of FVHs can serve as an imaging marker for the selection of endovascular therapy in acute occlusion of the MCA. A higher FVH-ASPECTS measured outside the DWI lesion is associated with good clinical outcomes in patients undergoing endovascular therapy, whereas the FVH-ASPECTS measured inside the DWI lesion is predictive of hemorrhagic transformation. It is noteworthy that the prognostic meanings of FVH before and after therapy are different. Jiang et al. (2019) assessed the relationship between functional outcomes and FVH before and after therapy and found that a group with good functional outcomes had higher FVH scores before therapy and lower FVH scores after therapy than a group with poor functional outcomes. FVHs before and after therapy were independently associated with functional outcomes. A high FVH score before therapy is regarded as a marker of good collateral status (Mahdjoub et al., 2018). However, the persistence of FVH after therapy is associated with persistent occlusion of a vessel and a poor functional outcome.

## Summary

Although the mechanism underlying FVH remains to be established, this phenomenon is associated with retrograde collateral blood flow and impaired hemodynamics in the ischemic territory due to intracranial stenoocclusive disease. FVH can be detected in acute infarction and chronic large-vessel occlusion. It is worth noting that FVH occurs in patients with TIA and might be correlated with clinical conditions such as atrial fibrillation and not only with large-vessel occlusion. However, it is still controversial whether FVH may serve as an imaging marker of hemodynamic impairment or good collateral status and may predict patients’ prognosis. Many factors should be considered when assessing the prognostic value of FVH, especially for symptom-to-imaging time and differences between before and after treatment. FVH may be an important neuroimaging marker, and radiologists and clinicians should be trained to look for its presence.

## Author Contributions

LZ and JC: conceptualization. LZ and HL: methodology and writing – original draft preparation. QW and MX: formal analysis and investigation. LZ and WW: writing – reviewing and editing. All authors contributed to the article and approved the submitted version.

## Conflict of Interest

The authors declare that the research was conducted in the absence of any commercial or financial relationships that could be construed as a potential conflict of interest.

## Publisher’s Note

All claims expressed in this article are solely those of the authors and do not necessarily represent those of their affiliated organizations, or those of the publisher, the editors and the reviewers. Any product that may be evaluated in this article, or claim that may be made by its manufacturer, is not guaranteed or endorsed by the publisher.
